# Supplementary material for: The interactome of intact mitochondria by cross-linking mass spectrometry provides evidence for coexisting respiratory supercomplexes
Source: Mol Cell Proteomics. 2017 Dec 8;17(2):216–32. doi: 10.1074/mcp.RA117.000470 (PMC5795388; doi:10.1074/mcp.RA117.000470)
Supplement: Supplemental Data [file supp_RA117.000470_133922_0_supp_23978_fzffwf.zip › spectra_annotation/mito_DR_spectra_annotation/202-1-7-1-3-1.pdf]

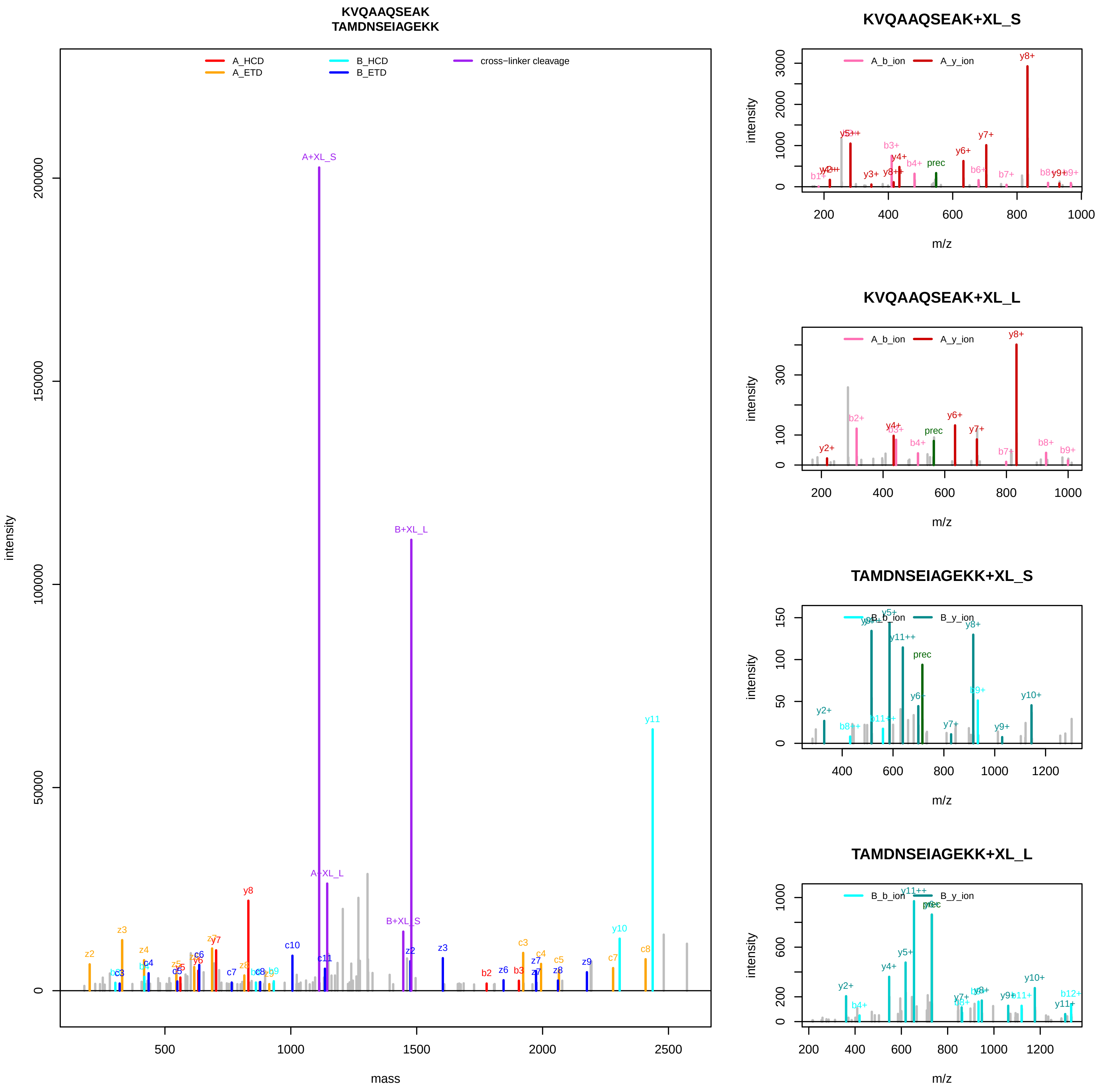
KVQAAQSEAK
TAMDNSEIAGEKK
A_HCD
A_ETD
B_HCD
B_ETD
cross−linker cleavage
A+XL_S
B+XL_L
A+XL_L
B+XL_S
y11
y10
y8
y7
y6
y5
c10
c11
c8
c7
c6
c5
c4
c3
z2
z3
z4
z5
z6
z7
z8
z9
b2
b3
b4
b9
intensity
mass
0
50000
100000
150000
200000
500
1000
1500
2000
2500
KVQAAQSEAK+XL_S
A_b_ion
A_y_ion
prec
m/z
KVQAAQSEAK+XL_L
TAMDNSEIAGEKK+XL_S
B_b_ion
B_y_ion
TAMDNSEIAGEKK+XL_L
